# Forest Nitrogen Dynamics in Response to Increasing Nitrogen Deposition: Comparing Above‐Canopy and Soil Fertilizations in a Mature Beech Forest

**DOI:** 10.1111/gcb.70534

**Published:** 2025-10-08

**Authors:** Alessandra Teglia, Cristiana Sbrana, Stefania Mattana, Andrea Scartazza, Matteo Bucci, Paola Gioacchini, Graziella Marcolini, Enrico Muzzi, Dario Ravaioli, Angela Ribas, Federico Magnani, Rossella Guerrieri

**Affiliations:** ^1^ Department of Agricultural and Food Science University of Bologna Bologna Italy; ^2^ National Research Council of Italy Institute of Agricultural Biology and Biotechnology (CNR‐IBBA) Pisa Italy; ^3^ Center for Ecological Research and Forestry Applications (CREAF) Barcelona Catalonia Spain; ^4^ Department of Agri‐Food Engineering and Biotechnology, Universitat Politècnica de Catalunya Castelldefels Spain; ^5^ National Research Council of Italy Research Institute on Terrestrial Ecosystems (CNR‐IRET) Pisa Italy; ^6^ National Biodiversity Future Center (NBFC) Palermo Italy; ^7^ BABVE, Edifici C Universitat Autònoma de Barcelona Bellaterra Spain

**Keywords:** ectomycorrhizal root tip, European beech, forest nitrogen cycling, microbial functional genes, nitrogen manipulation experiment, soil microbes, stable nitrogen isotopes

## Abstract

Nitrogen (N) fertilization experiments provide critical insights into how increasing N deposition alters the balance between N retention and saturation in forest ecosystems. However, most studies have considered soil N applications, overlooking tree canopy‐atmosphere interactions, leading to an incomplete understanding of the fate of N in forests. We investigated ecosystem N dynamics 4 years after the establishment of a nitrogen manipulation experiment in a beech forest, involving Control (N0), 30 kg N ha^−1^ y^−1^, distributed to soil (N30) and above tree canopies (N30A), and 60 kg N ha^−1^ y^−1^ applied to the soil (N60). We assessed N concentration and δ^15^N across forest compartments (leaves, fine roots, ectomycorrhizal root tips, soil, and litter) and quantified microbial functional genes related to soil N processes. N concentrations were minimally affected by treatments, whereas δ^15^N increased along compartments, particularly in the N60, indicating enhanced soil N losses. Both N30 and N60 increased N concentrations and δ^15^N values in ectomycorrhizal root tips (ERT) and soil, suggesting enhanced fungal N immobilization but limited transfer to the host plants. In contrast, N30A led to ^15^N depletion in fine roots and litter, reflecting stronger plant reliance on ectomycorrhizal activity and potential alterations in litter quality, which may inhibit decomposition. Soil nitrifiers and denitrifiers were abundant, regardless of the treatments. Our findings highlight the need for future experiments to simulate realistic N deposition scenarios, including canopy interactions, to better understand ecosystem N dynamics and forest responses under global change.

## Graphical abstract

This study explored how nitrogen (N) addition affects a mature beech forest after 4 years of fertilization, comparing soil and above‐canopy fertilization to mimic increased atmospheric nitrogen. While total N levels changed little, the N isotope data (d^15^N) showed contrasting effects: soil fertilization enhanced N loss, whereas canopy fertilization promoted N retention and stronger interactions between plants and fungi. These results emphasize the importance of including canopy N inputs in future studies to better understand ecosystem N dynamics and plant–microbe interactions under changes in N deposition.

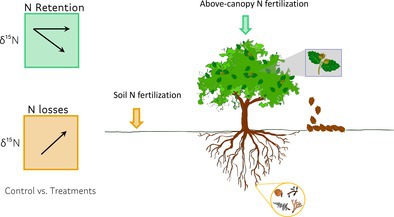

## 1 Introduction

Reactive nitrogen (N) compounds in the atmosphere, and their input to terrestrial ecosystems via wet and dry deposition, have substantially increased in recent decades, due to anthropogenic activities, such as intensive use of fertilizer in agriculture, livestock, and fossil fuel combustion (Galloway et al. 2004). As a result, we have observed significant alterations of processes underpinning the N cycle (Steffen et al. 2015) and, consequently, of N availability in temperate forest ecosystems, which are commonly N‐limited (Galloway et al. 2008; Rennenberg and Dannenmann 2015). The increase in atmospheric N input can enhance photosynthesis (Janssens and Luyssaert 2009), tree growth (Etzold et al. 2020), and forest carbon sequestration (Magnani et al. 2007; Wang et al. 2017; Schulte‐Uebbing and de Vries 2018; Du and de Vries 2024). However, if the extra input of N exceeds the actual demand by trees and microbial communities, the ecosystem can experience saturation (Aber et al. 1998), fostering N losses through leaching, denitrification, and ammonia volatilization, with adverse effects on forest productivity and functioning (Takahashi et al. 2020; Etzold et al. 2020). The extent and the timing of these negative effects, however, depend on the status of the ecosystem and its capacity for N retention (Aber 2002; Du and de Vries 2025) as well as tree species composition (Oulehle et al. 2025).

An increase in atmospheric N input to forest ecosystems leads to elevated rates of internal N cycling, which can be captured by measuring changes in ^15^N natural abundance in forest samples (Craine et al. 2015). Key soil processes, including decomposition, mineralization, nitrification, denitrification, and ammonia volatilization, preferentially discriminate against the heavier isotope, ^15^N (Hobbie and Ouimette 2009; Högberg 1997; Hobbie and Colpaert 2003). As a result, the products of these processes are relatively depleted in ^15^N, while the substrates from which they originated become relatively enriched in ^15^N (Peterson and Fry 1987; Yoneyama 1996). Thus, at elevated N cycling rates, soil‐available N tends to be enriched in ^15^N, since the lighter isotope, ^14^N, preferentially exits the ecosystem through N loss pathways (Högberg 1990, Högberg 1997). As plants take up the relatively ^15^N‐enriched N forms available in the soil, they exhibit more positive δ^15^N values, reflecting the isotopic composition of the N they assimilate (Templer et al. 2007). Several studies demonstrated a robust positive relationship between foliar δ^15^N and soil N concentration and N process rates (Emmett et al. 1998; Meints et al. 1975; Högberg 1990; Gebauer and Schulze 1991; Craine et al. 2015). Although the inherent complexity in interpreting data from ^15^N natural abundances in terrestrial ecosystems, they are commonly employed as tracers or proxies for ecological processes (Craine et al. 2015).

Over the last few decades, numerous N manipulation experiments have been conducted to understand the impacts of increasing N deposition on forest ecosystems (Bebber 2021). However, in the “old‐generation” fertilization experiments, the applied N doses often greatly exceeded the actual atmospheric deposition (de Schrijver et al. 2011; Hyvönen et al. 2008; Billow et al. 1994; Kaakinen et al. 2009; Bebber 2021). Moreover, the increase in atmospheric N input was typically simulated by direct soil applications, excluding a priori potential interactions between atmospheric N and tree canopies. Indeed, there is mounting evidence that tree canopies can intercept a substantial amount of atmospheric N deposition (Sievering et al. 2007; Gaige et al. 2007; Dail et al. 2009, Guerrieri et al. 2021 and reference therein). While part of the intercepted N may undergo transformation or volatilization, being lost from the ecosystem, a portion of it could be directly assimilated by foliage, thus being readily available for plant N metabolism (Sparks 2009; Ferraretto et al. 2022), or it can be used by microbes hidden in the phyllosphere (Guerrieri et al. 2021, 2024). Therefore, direct‐to‐soil N additions might have overlooked important processes and inaccurately simulated the fate of N deposition and its impact on forest biogeochemical processes (Bortolazzi et al. 2021; Da Ros et al. 2023) as well as soil microbial diversity and community compositions (Liu et al. 2022). Indeed, most of the soil N manipulation experiments, often applying doses up to 10 times higher than the ambient N deposition (Bebber 2021), reported evidence of N saturation with increasing N doses applied, as detected by using δ^15^N in soil and foliar samples (Nadelhoffer et al. 1999; Magill et al. 2004). An increase in NO_3_^−^ leaching at European forest sites with N deposition above 10 kg ha^−1^ y^−1^ has been reported (Gundersen et al. 2006), but there is no clear evidence that this loss pathway increases over time under elevated N deposition or reliably indicates N saturation (Du and de Vries 2025). In contrast, recent analyzes (including also δ^15^N in foliar samples) have identified pervasive N limitation in forests (Craine et al. 2018; Mason et al. 2022), which may constrain the expected CO_2_ fertilization effect on carbon sequestration (Zaehle 2013; Terrer et al. 2019; Wang et al. 2020). As the impacts of N deposition when moving along the continuum atmosphere–tree–soil involve intricate dynamics, it is crucial to elucidate how increasing levels of N deposition alter plant–soil interactions, to achieve a greater understanding of the influence of N deposition on the interplay between N and carbon cycling in forest ecosystems.

Here we investigated the effect of simulated increase in N deposition on ecosystem N dynamics, with particular reference to retention versus saturation trajectories. By applying both direct‐to‐soil and above‐canopy N fertilization in a mature deciduous forest, we could assess whether soil fertilizations overestimate effects of N deposition on ecosystem N dynamics. We tested the following hypotheses: (1) N additions would increase N availability, regardless of the approach (above‐canopy vs. direct‐to‐soil fertilizations) and doses applied; (2) direct‐to‐soil fertilization would enhance soil N processes, leading to higher N concentration and δ^15^N values across ecosystem compartments, as well as microbial functions associated with N loss pathways (denitrification); (3) the two fertilization approaches may diverge in terms of tree N‐uptake strategies, with less conservative N acquisition under direct‐to‐soil fertilization, due to greater N availability. To test our hypotheses, we combined the measure of N concentration and δ^15^N across different forest compartments (i.e., leaves, fine roots, ectomycorrhizal root tips (ERT), soil, and decomposed and undecomposed litter) with molecular analyzes on soil microbes to assess potential changes in the functional genes associated with key N processes under the simulated increase in atmospheric N inputs.

## 2 Material and Methods

### 2.1 Experimental Site

The study site is located within a dense, single‐layered beech ( *Fagus sylvatica* L.) forest, in Pian del Cansiglio (46° 3′ 19" N 12°22′51" E, 1100 m a.s.l.), in the Eastern Italian Alps, approximately 50 km northeast of the Po valley. The soil is calcareous, nutrient‐rich, and relatively moist Haplic Luvisol (WRB). The climate is oceanic, with a mean annual temperature of 7.2°C and a mean annual precipitation of 1767 mm. The forest stand has a tree density of 168 trees per hectare, with the trees aged between 130 and 140 years (Teglia et al. 2022). The experimental site is located near the VEN1 long‐term monitoring plot within the European ICP Forests and Italian CONECOFOR networks (Marchetto et al. 2014; Cecchini et al. 2021). The average annual N deposition from 1998 to 2017 is 17.7 kg ha^−1^ y^−1^ (bulk deposition) and 12.6 kg N ha^−1^ y^−1^ (throughfall), with N deposition in the form of NO_3_^−^ showing a significant decreasing trend, while no significant changes were observed in the case of N deposition in the form of NH_4_^+^ (Cecchini et al. 2021).

### 2.2 Experimental Design

Four N addition treatments were established in 2015 (and are still ongoing): control (N0, receiving only ambient deposition), soil additions of 30 (N30) and 60 kg N ha^−1^ year^−1^ (N60), and above‐canopy addition of 30 kg N ha^−1^ year^−1^ (N30A). The experiment followed a completely randomized design with three replicate plots per treatment. Plots have a square shape (30 × 30 m) for the N0, N30, and N60 treatments, and a circular shape (20 m radius) for the N30A treatment. To prevent any lateral contamination effects, intensive sampling and tree measurements were conducted within the central area (15 × 15 m) of each plot.

The N30 and N30A treatments of 30 kg N ha^−1^ y^−1^ were considered for mimicking the effects of a realistic increase in N deposition, which was slightly above the ambient deposition in the area, that is, 20 kg N ha^−1^ y^−1^ when the experiment started in 2015. Moreover, the dose of 60 kg N ha^−1^ y^−1^ was intended to potentially drive the ecosystem toward a state of N saturation.

N was applied using an ammonium nitrate (NH_4_NO_3_) solution, with an isotopic composition of the fertilizer applied of −0.06‰ ± 0.52‰ (*n* = 4), in line with values reported in the literature (Högberg et al. 2024). For direct‐to‐soil treatments (N30 and N60), a backpack sprayer was utilized to add the solution (35.1 g N L^−1^). In the case of above‐canopy fertilization (N30A), the solution (4.5 g N L^−1^) was dispersed above the tree canopy using a circular sprinkler installed on the central tree within the plots. N addition started in May 2015, and since then, treatments have been performed three times per year during the growing season (June, July, and September).

While we do not have historical measurements of δ^15^N values of N deposition, preliminary measurements conducted in July 2021 in a control plot showed that δ^15^N values of NH_4_^+^ and NO_3_^−^ extracted from bulk deposition (1 sample collected in an open space outside the forest, NH_4_^+^ and NO_3_^−^ concentrations: 0.72 and 0.49 mg L^−1^, respectively) were + 8.31‰ and −1.66‰, respectively, and those from throughfall water (1 sample, NH_4_^+^ and NO_3_^−^ concentrations: 0.12 and 0.57 mg L^−1^) were +2.89‰ and −2.25‰, respectively. These values fall within the range reported in the literature (−15‰ and +15‰ compared to 0‰ of atmospheric N_2_, Kendall et al. 2007). Although limited, these measurements suggest that the applied dose and the fertilization approaches (above‐canopy vs. soil applications), rather than the isotopic signature of the fertilizer (which is closer to the δ^15^N of the atmospheric N_2_), likely had a greater influence on ecosystem processes.

### 2.3 Sampling Along the Forest Profile

The sampling campaign was conducted in July 2018, 4 years after the treatments started. Fresh, sun‐exposed leaves were sampled from three trees within each plot (*n* = 36; see Teglia et al. 2022 for details). Leaves were washed, oven‐dried at 60°C until constant weight, and ground into powder. Within the intensive area of each plot, two trees were targeted to collect fine roots, soil, and differentiate organic layers (partially undecomposed litter—OF, and decomposed litter—OH). Specifically, at 1 m distance from each of the two selected trees, three equidistant points were identified for root and soil sampling. Organic layers (OF and OH, 1 cm depth) and soil cores (5 cm diameter and 10 cm depth) were collected and pooled for each tree, leading to a total of *n* = 24 samples across treatments. Fine roots below the litter layer were collected around the selected tree and pooled together, creating a comprehensive sample representative of the tree's fine root system, with a total of *n* = 24 samples across treatments. Finally, in autumn, beech leaf litter and fruits were collected from the five collectors located in the central area of the plot and pooled together (one sample per plot, *n* = 12).

### 2.4 Sample Preparation and Chemical Analyses

In the laboratory, litter layers (OH and OF) and an aliquot of soil subsamples were sieved with a 2 mm mesh sieve, oven‐dried at 60°C until a constant weight was reached, and then were ground into a fine powder. The remaining soil samples were kept at −20°C for microbial genetic analysis. Fine roots (< 2 mm in diameter) were detached from coarse roots, cleaned with sterile distilled water over a 100 μm mesh sieve, and then stored at −20°C until analysis. Fine roots were placed in Petri dishes containing distilled water to distinguish ERT from ectomycorrhizal‐free fine roots. Under a stereomicroscope (Carl Zeiss 475003‐9902), meticulous removal of residual soil and organic particles was conducted using fine‐tipped forceps. Dead and dry root fragments and tips (black withered roots showing turgor loss) were discerned from living ones and were excluded from further processing. The ERT and the ectomycorrhizal‐free root tips were counted, allowing the calculation of the ERT percentage (fraction of ERT to total viable root tips) for each sample. Approximately 62% were found to be colonized and there were no significant differences across N treatments. In the next step, the fine roots were sectioned into pieces using a sterile blade, while all ERTs were carefully separated just above the fungal mantle to produce ERT samples following the protocol described in Scartazza et al. (2023). Both ERT and fine roots samples were dried at 60°C until reaching a constant weight and ultimately ground into a fine powder. Fruit capsules were discarded and only seeds were dried at 60°C until constant weight, weighed, and ground into a fine powder.

The total N concentration (%N) and the stable N isotopic composition (δ^15^N) were analyzed in a continuous flow‐isotope ratio mass spectrometry (CF‐IRMS) using an elemental analyzer (Flash 2000, Thermo Fisher Scientific, USA) coupled with an isotope ratio mass spectrometer (Delta V Advantage Thermo Fisher Scientific, USA). Isotopic values are expressed in the delta (δ) notation as a relative deviation from the international standard (Fry 2006) according to the equation:

(1) $$\delta^{15}N\left(‰\right)=\left(\frac{R_{\text{sample}}}{R_{\text{standard}}}-1\right)\times 1000$$

where *R*_sample_ and *R*_standard_ are the ^15^N and ^14^N isotope ratios of the sample and the international standard (i.e., the atmospheric N_2_: 0.0036782), respectively. Analytical precision was ±0.2‰ based on replicate analysis of the reference material wheat flour standard (OAS Cat No. IVA33802157). Raw data of δ^15^N and %N can be found in Teglia et al. (2025).

### 2.5 Quantification of Functional Genes Related to Soil N Processes

Microbial DNA was extracted from 0.25 g of soil using the DNeasy PowerSoil Kit (Qiagen Inc., USA) according to the manufacturer's protocol. DNA concentration was determined with a Qubit 3.0 Fluorometer (Invitrogen, Carlsbad, CA, USA) using the high‐sensitivity (HS) double‐stranded DNA assay. Quantitative PCR (qPCR, real‐time PCR) was performed to quantify functional genes involved in key soil nitrogen processes. Specifically, we targeted genes encoding the ammonia monooxygenase subunit A of bacteria (amoA AOB) and archaea (amoA AOA), and the nitrite oxidoreductase subunit B of bacteria (nxrB), which catalyze the oxidation of ammonia to nitrite and nitrite to nitrate, respectively. Moreover, we quantified bacterial genes involved in denitrification, including those encoding nitrite reductases (bacterial nirS and bacterial and fungal nirK), nitric oxide reductase (bacterial qnorB), and nitrous oxide reductase (bacterial nosZ). Finally, the functional gene encoding dinitrogenase reductase involved in nitrogen fixation (bacterial nifH) was also quantified. See Supporting Information for details on the methodology (Table S1). Gene abundances were expressed on a log10 scale as copies per gram of soil, normalized to both microbial DNA extracted and soil dry weight. Raw data can be found in Teglia et al. (2025).

### 2.6 Assessing Differences Between Treatments in N Uptake and Soil–Plant N Pathways

The enrichment factor, often used as an indicator of ecosystem N status (Emmett et al. 1998; Amundson et al. 2003), was calculated as a difference between the leaf and soil δ^15^N values (EF = δ^15^N_leaves_—δ^15^N_soil_).

**FIGURE 1:**
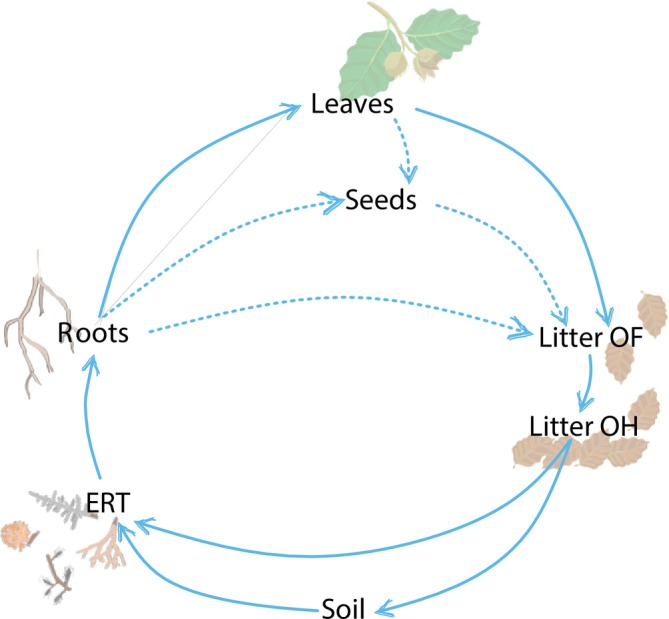
Simplified scheme showing soil–plant N pathways, which include the forest ecosystem components measured in this study: Soil, ectomycorrhizal root tips (ERT), fine roots (roots), leaves, seeds, litter OF, and litter OH. Solid lines indicate pathways considered in our analyzes, while dashed lines refer to other relevant pathways that may occur but that were not directly addressed here.

The evaluation of δ^15^N differences (Δδ^15^N, where the Δ indicates the offset) between close compartments within the forest was conducted using the simplified N uptake and within‐plant N pathways as summarized in Figure 1. Specifically, we calculated the offsets between consecutive compartments to assess the influence of N addition on N uptake and translocation processes. Positive values denote an enrichment in ^15^N from the previous to the subsequent compartment, while the opposite is true in the case of negative offset values. The calculations were performed using the following equations:

(2) $${\Delta \delta }^{15}N_{\left(\text{soil}-\mathrm{ERT}\right)}=|\delta^{15}N_{\text{soil}}|-|\delta^{15}N_{\mathrm{ERT}}|$$

(3) $${\Delta \delta }^{15}N_{\left(\text{LitterOH}-\mathrm{ERT}\right)}=|\delta^{15}N_{\text{LitterOH}}|-|\delta^{15}N_{\mathrm{ERT}}|$$

(4) $${\Delta \delta }^{15}N_{\left(\mathrm{ERT}-\text{Fine roots}\right)}=|\delta^{15}N_{\mathrm{ERT}}|-|\delta^{15}N_{\text{Fine roots}}|$$

(5) $${\Delta \delta }^{15}N_{\left(\text{Fine roots}-\text{Leaves}\right)}=|\delta^{15}N_{\text{Fine roots}}|-|\delta^{15}N_{\text{Leaves}}|$$

(6) $$\Delta \delta^{15}N_{\left(\text{Leaves}-\text{LitterOF}\right)}=|\delta^{15}N_{\text{Leaves}}|-|\delta^{15}N_{\text{LitterOF}}|$$

(7) $$\Delta \delta^{15}N_{\left(\text{LitterOF}-\text{LitterOH}\right)}=|\delta^{15}N_{\text{LitterOF}}|-|\delta^{15}N_{\text{LitterOH}}|$$

(8) $$\Delta \delta^{15}N_{\left(\text{LitterOH}-\text{Soil}\right)}=|\delta^{15}N_{\text{LitterOH}}|-|\delta^{15}N_{\text{soil}}|$$

The seeds compartment was excluded from offset calculation due to the limited number of replicates.

For evaluating differences among treatments for N concentration and δ^15^N measured in each compartment, all data were averaged within the treatment, considering individual trees as replication units, except for seeds, where the plot served as the replication unit. Dixon's test was utilized to detect outliers, which were then excluded from the analyzes if identified; moreover, Bartlett's test was employed to assess the homogeneity of variance before the analysis. One‐way analysis of variance (ANOVA) was employed to detect differences in mean N concentration (%), δ^15^N (‰), Δδ^15^N (‰), and functional genes among treatments (N0, N30A, N30, N60) within each forest compartment. A posteriori separation of the means was performed by the Student Newman–Keuls test (SNK). The significance level was set at *p* ≤ 0.05, with results at *p* ≤ 0.01 and *p* ≤ 0.001 also explicitly reported when applicable. All statistical analyzes were conducted using R open‐source software (R Core Team 2017) and the following specific packages: lavaan (Yves 2012), agricolae (de Mendiburu and Yaseen 2020), Outliers (Komsta 2011), easyanova (Arnhold 2013), ggplot2 (Wickham 2016), Tidyverse (Wickham et al. 2019).

## 3 Results

### 3.1 Relationships Between N Concentration and δ^15^N Across Forest Samples

**FIGURE 2:**
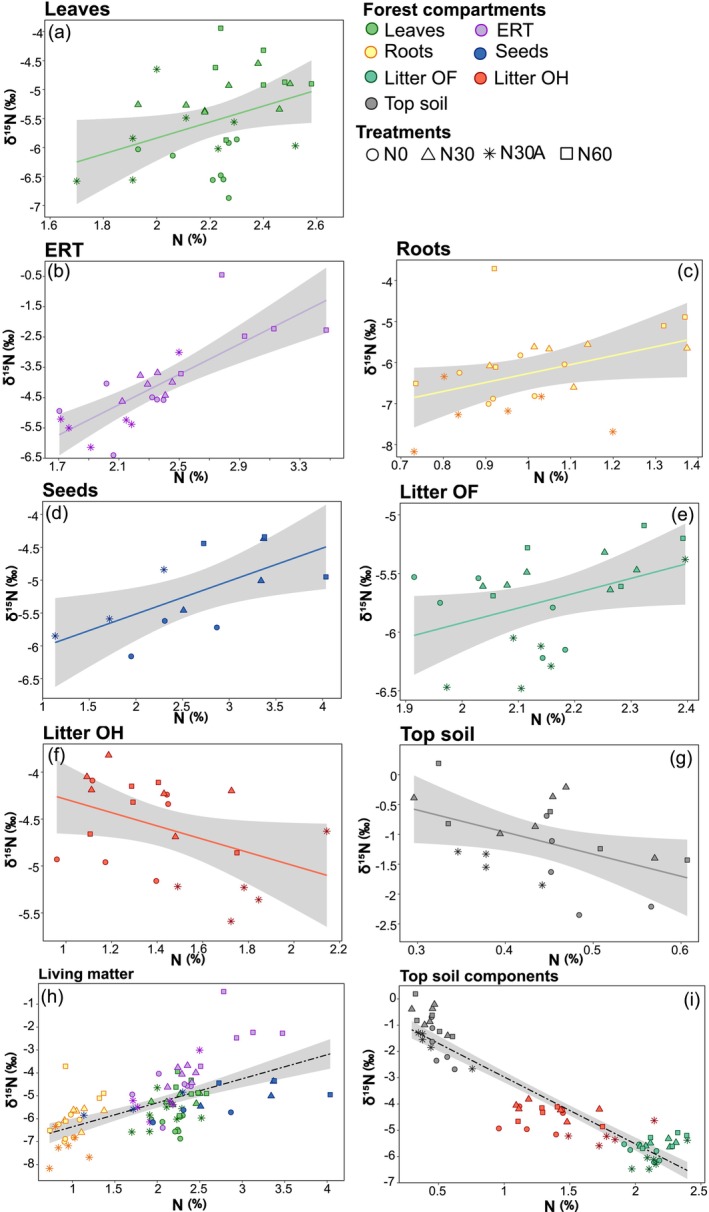
Linear relationships between N isotope composition (δ^15^N) and N concentration in different forest compartments: Leaves (a), ectomycorrhizal root tips (ERT) (b), ectomycorrhizal‐free fine roots (roots, c), seeds (d), litter OF (e), litter OH (f), mineral soil (top soil, g), all plant organic matter together (h), and soil components (i). Each point corresponds to a measurement from a single tree, except for seeds where the sample units were the plots. Different shapes and colors indicate the treatments applied and forest compartments, respectively, as reported in the legend. Key statistical parameters from regression analyzes are: (panel a) adjusted *R* ^2^ = 0.11, intercept = −8.59, slope = 1.38, *p* < 0.05; (panel b) adjusted *R* ^2^ = 0.60, intercept = −9.99, slope = 2.5, *p* < 0.001; (panel c) adjusted *R* ^2^ = 0.13, intercept = −8.44, slope = 2.18, *p* = 0.054; (panel d) adjusted *R* ^2^ = 0.38, intercept = −6.52, slope = 0.50, *p* < 0.05; (panel e), adjusted *R* ^2^ = 0.14, intercept = −8.45, slope = 1.26, *p* < 0.05; (panel f), adjusted *R* ^2^ = 0.14, intercept = −3.58, slope = −0.71, *p* = 0.050; (panel g) adjusted *R* ^2^ = 0.42, intercept = 0.92, slope = −4.69, *p* < 0.001; (panel h) adjusted R^2^ = 0.35, intercept = −7.40, slope = 1.05, *p* < 0.001; (panel i) adjusted R^2^ = 0.86, intercept = −0.42, slope = −2.55, *p* < 0.001.

A significant positive correlation between δ^15^N and N concentration was observed across all studied compartments (adjusted *R* ^2^ = 0.353, *p* < 0.001, Figure 2, panel h), except for fine roots (*p* = 0.054; Figure 2, panel c), while δ^15^N and N in soil were negatively correlated (adjusted *R* ^2^ = 0.80, *p* < 0.001, Figure 2, panel i). Within each forest compartment, the strongest relationship was observed in ERT (adjusted *R* ^2^ = 0.60, *p* < 0.001; Figure 2, panel b), whereas mineral soil N exhibited the strongest negative relationship (adjusted *R* ^2^ = 0.42, *p* < 0.001; Figure 2, panel g). Notably, we did not observe a difference among treatments for the relationships between δ^15^N and N across the various compartments.

### 3.2 Differences Among Treatments for N Concentration and δ^15^N Values Across Forest Compartments

**FIGURE 3:**
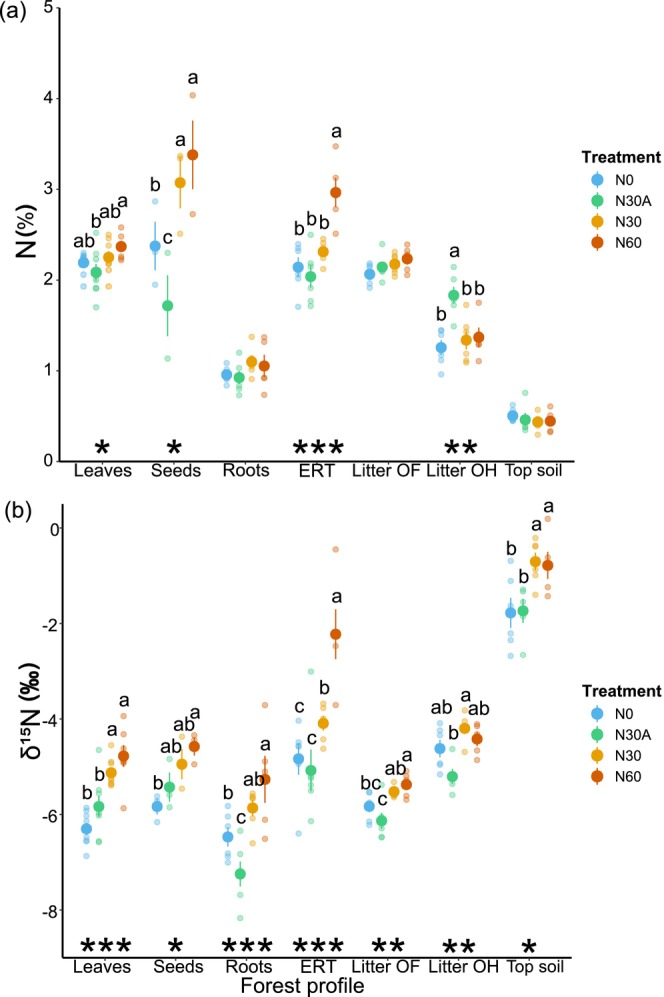
Mean values (± standard errors) of N concentration (%) (panel a) and nitrogen isotope composition (δ^15^N, ‰) (panel b) across different forest compartments for each of the four treatments (indicated with different colors). Different letters indicate significant differences (*p < 0.05, **p < 0.01, ***p < 0.001) in mean values among treatments within each compartment, based on ANOVA followed by the SNK test. ERT indicates ectomycorrhizal root tips (ERT), while root indicates ectomycorrhizal‐free fine roots.

Four years after the onset of fertilization, we did not detect any significant difference in N concentration between the control and all N treatments in the case of leaves, fine roots, litter OF, and soil (Figure 3, panel a). Notably, N30 and N60 had a positive effect on N concentration in seeds (+29.4% for N30 and +42.3% for N60 vs. N0, *p* < 0.05, Figure 3 panel a) and in ERT (+38.4% in N60 compared to N0, *p* < 0.001, Figure 3 panel a). Conversely, the N30A affected N concentration only in the case of the litter OH, which increased by 45.9% compared to the control (*p* < 0.01, Figure 3 panel a).

The direct‐to‐soil fertilization treatments substantially affected N isotopic composition across the investigated compartments. Remarkably, we observed a significant enrichment in ^15^N in all analyzed forest compartments in the N60, with the only exception of the litter OH (Figure 3, panel b). The substantial increase in δ^15^N was particularly evident in ERT, leaves, and seeds, showing increases of 53.9%, 24.2%, and 21.5%, respectively, compared to the control. Similar results were also observed in N30, although differences from N0 were statistically significant only for leaves (+18.6%), ERT (+15.3%), and soil (+60.3%). Conversely, the effect of N30A on δ^15^N was less pronounced (Figure 3, panel b). Similarly to what was observed in the case of N30 and N60, the above‐ground forest components (i.e., leaves and seeds) were more enriched in ^15^N compared to the control, though the difference was not significant. In the case of below‐ground compartments (i.e., litter layers, ERT, and fine roots), samples were generally more depleted in ^15^N (i.e., more negative δ^15^N values) in N30A than N0, except for soil δ^15^N, where the mean values were similar between N0 and N30A. A significant difference between N30A and N0 was observed only in the case of δ^15^N values measured in fine roots (−0.78‰, Figure 3 panel b). Forest compartments in N30A were generally less enriched in ^15^N compared to N30, except for seeds and litter OF.

### 3.3 Quantification of Functional Genes Related to Soil N Processes

**FIGURE 4:**
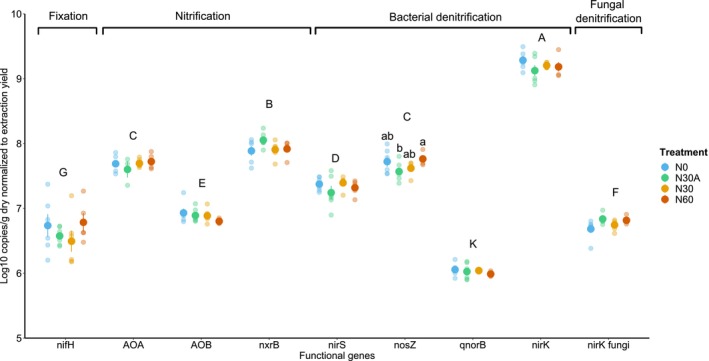
Abundance of microbial functional genes involved in key soil N processes: NifH (nitrogenase gene), AOA and AOB (archaeal and bacterial amoA ammonia monooxygenase genes), nxrB (nitrite oxidoreductase genes), nirS and nirK (nitrite reductase genes), nosZ (nitrous oxide reductase genes), qnorB (nitric oxide reductase), and nirK fungi (fungal nitrite reductase genes). Treatments are displayed in different colors. Capital letters indicate statistical differences across soil functional genes, while lowercase letters denote differences within treatments for each functional gene (based on SNK test).

Treatments, regardless of the N fertilization approaches, did not significantly alter the abundance of microbial functional genes involved in soil N processes (NFGs) (Figure 4), except for nosZ (*p* < 0.05), which, however, did not show a clear treatment effect.

Regardless of the treatment, we found a significant (*p* < 0.001) difference among microbial functionality related to N processes (Figure 4). Archaeal nitrifiers (amoA AOA = 7.68 ± 0.1 log_10_ copies/g) were significantly higher than bacterial nitrifiers (amoA AOB = 6.88 ± 0.1 log_10_ copies/g). Moreover, bacterial nitrifiers involved in the nitrite to nitrate step of nitrification (nxrB) were significantly higher than those bacterial involved in the first step of nitrification (amoA AOB). Except for qnorB, all the bacterial denitrifiers (nirK = 9.20 ± 0.2, nirS = 7.33 ± 0.2, and nosZ = 7.67 ± 0.2—log_10_ copies/g) were more abundant than fungal denitrifiers (nirK = 6.77 ± 0.3 log_10_ copies/g; Figure 4). The abundance of bacterial nirK genes was the highest among all the investigated soil functional genes. Finally, the functional gene related to N_2_ fixation (nifH) was the least abundant (6.65 ± 0.3 log_10_ copies/g) in the soil (Figure 4).

### 3.4 Enrichment Factor and Offset in δ^15^N Between Compartments

The Enrichment factor (EF) exhibited a range between −4.52‰ and −3.18‰ (Table 1). Although the treatments generally displayed higher EF values than the N0 plots, this increase was not statistically significant. Nevertheless, N fertilizations significantly altered the relative ^15^N enrichment between compartments along the key pathways of tree‐soil N interactions, that is, the offset between compartments (Δδ^15^N) (Table 1). In comparison to soil and litter OH, ERT tissues exhibited a greater enrichment in ^15^N in the N60 treated plots. In the control plots, Δδ^15^N_(LitterOH‐ERT)_ values were nearly zero (−0.22‰ ± 0.3‰), indicating a relatively consistent δ^15^N between the two compartments. Conversely, in the N60 plots, the Δδ^15^N_(LitterOH‐ERT)_ was 2.19‰ ± 0.5‰, indicating that ERT tissues were more enriched in ^15^N than the organic substrate. A contrasting pattern emerged in the pathway from ERT to fine root tissues, where the depletion in ^15^N from ERT to the fine roots compartment was notably higher in N60 plots (−3.04‰ ± 0.2‰) compared to N0 plots (−1.64‰ ± 0.2‰). While a ^15^N enrichment from fresh leaves to partially decomposed litter (OF) was evident in N0 plots (0.48‰ ± 0.2‰), the Δδ^15^N_(Leaves‐LitterOF)_ in N treatments (N30A, N30, and N60) significantly differed from the control (*p* < 0.001). Indeed, negative Δδ^15^N values were observed in fertilized plots, meaning a consistent depletion in ^15^N in litter OF compared to fresh leaves, which increased when moving from above‐canopy (−0.37‰ ± 0.2‰) to soil treatments (−0.46‰ ± 0.1‰, and −0.71‰ ± 0.2‰ for N30 and N60, respectively). Additionally, only the N30A significantly affected the Δδ^15^N_(Fine roots‐Leaves)_ values (1.48‰ ± 0.2‰, *p* < 0.01), which denoted a greater ^15^N enrichment in leaves from fine roots compared to the N30, N60, and N0 plots.

**TABLE 1:** Enrichment factor (EF) and offset (Δδ^15^N) of δ^15^N values between subsequent forest compartments.

	N fertilization treatments				
	N0	N30A	N30	N60	*p*
**Enrichment factor (‰)**					
*EF*	−4.52 ± 0.3	−3.87 ± 0.3	−4.36 ± 0.3	−3.81 ± 0.4	ns
**Δδ** ^**15**^ **N (‰)**					
*Soil—ERT*	−3.06 ± 0.4 b	−3.32 ± 0.6 b	−3.39 ± 0.3 b	−1.44 ± 0.7 a	< 0.05
*LitterOH—ERT*	−0.22 ± 0.3 b	−0.29 ± 0.3 b	0.10 ± 0.2 b	2.19 ± 0.5 a	< 0.001
*ERT—Fine roots*	−1.64 ± 0.2 a	−2.17 ± 0.4 a	−1.77 ± 0.2 a	−3.04 ± 0.2 b	< 0.05
*Fine roots—Leaves*	0.17 ± 0.2 b	1.48 ± 0.2 a	0.80 ± 0.1 b	0.37 ± 0.6 b	< 0.01
*Leaves—Litter OF*	0.48 ± 0.2 a	−0.37 ± 0.2 b	−0.46 ± 0.1 b	−0.71 ± 0.2 b	< 0.001
*LitterOF—LitterOH*	1.21 ± 0.2	0.86 ± 0.2	1.33 ± 0.1	0.95 ± 0.2	ns
*LitterOH‐Soil*	2.84 ± 0.2	3.49 ± 0.2	3.50 ± 0.2	3.54 ± 0.2	ns

*Note:* The enrichment factor is calculated as the difference between δ^15^N in leaf and δ^15^N in soil. Each value represents the mean ± standard error (*n* = 6). Letters indicate differences within the rows (based on the SNK test).

## 4 Discussion

### 4.1 Did N Additions (Regardless of the Approach) Increase N Availability?

Four years of N fertilization treatments, regardless of the approach (above‐canopy or direct‐to‐soil), did not substantially change the N concentrations in the majority of the compartments analyzed (Figure 3), thus not confirming our first hypothesis. Indeed, we did not detect a significant increase in N concentration in leaves, fine roots, litter, or soil, which is consistent with results from similar manipulation experiments in the literature (Zhang et al. 2015; Da Ros et al. 2023) applying low N doses to simulate a more realistic increase in N deposition. Indeed, Da Ros et al. (2023) reported no differences in N content across forest ecosystem compartments following the addition of 20 kg N ha^−1^y^−1^, applied both above and below the canopy in a temperate deciduous forest. Similarly, Zhang et al. (2015) did not detect differences in leaf N content after the addition of 25 or 50 kg N ha^−1^y^−1^ either above or below the canopy in a subtropical deciduous forest.

We expected to observe a more rapid response of N concentration in the tree compartments—such as leaves, seeds, and fine roots—in the case of N30A compared to the N0 and N30 treatments, due to potential canopy N uptake (Ferraretto et al. 2022). In a forest‐scale aerial N spraying experiment using a helicopter, 70% of the applied N was retained by tree canopies (Gaige et al. 2007). However, this did not necessarily result in a direct plant uptake, as most of the retained N was recovered on plant surfaces, such as twigs, branches, and stems, thus potentially remaining unavailable for plant uptake for several years (Dail et al. 2009).

An interesting trend emerged in the case of seed N concentrations. While N concentration in seeds was higher in N30 and N60 compared to N0, the opposite was observed in the case of N30A (lower N concentration compared to N0). In N30 and N60, an increase in fruit production was also observed (data not shown). This, coupled with the higher N concentration found in seeds compared to leaves, might suggest that the highly nutrient‐demanding process of fruit production could take advantage of the additional N supply (Han et al. 2017). We acknowledge that our results are based on a limited number of replicates (three per treatment) for seed samples. Nevertheless, they provide insights into the effects of N deposition on nutrient allocation dynamics during seed production, which should be explored further.

While we expected a general increase in N concentration across all belowground compartments, we did not anticipate that this would be observed only in the ERT compartment, and only under the highest dose of soil fertilization (N60). The lack of substantial variation in N concentration within both tree and soil compartments may be partly attributed to the eutrophic condition of the forest site, where N is not a growth‐limiting factor (Teglia et al. 2022). As a result, the relatively low doses applied for 4 years did not significantly alter the N concentrations across the forest compartments.

### 4.2 δ^15^N as a Better Indicator Than N Concentration of Changes in N Dynamics Induced by Above‐Canopy and Soil Fertilization

While the absence of substantial changes in N concentrations introduces uncertainty regarding potential alterations in ecosystem N dynamics, a clearer picture emerged when looking at the δ^15^N values across the different forest compartments (Figure 3). Under increasing levels of N availability (for instance under increasing N deposition), plant tissues are expected to show higher δ^15^N values (Templer et al. 2007; Craine et al. 2015). Here, we observed a positive correlation between δ^15^N and N concentration in plant compartments irrespective of the treatment applied (Figure 2), thus confirming δ^15^N as a valuable indicator for depicting changes in ecosystem N dynamics.

N isotopic composition consistently increased across forest compartments in soil fertilization vs. above canopy fertilization and control, particularly in the case of N60. Does this suggest that soil fertilization is enhancing nitrification and hence N loss from the system via denitrification? While direct measurements of nitrification and denitrification were not carried out at the site, we employed molecular analyzes to quantify the abundance of microbial functional genes directly involved in these processes (Figure 4). This indirect approach allows us to assess whether various fertilization approaches resulted in alteration of microbial functionality related to soil N loss pathways.

Analysis of the NFGs data revealed that the abundance of microbes associated with key N processes, that is, N fixation, nitrification, and denitrification, remained largely unaffected by the various N treatments applied. Nevertheless, the abundances of NFGs reflected patterns typically observed in N‐rich forest soils, characterized by low soil C/N ratios (mean value in the control plots is 12.12 in our study site) and exhibiting a high rate of N cycling (Tang et al. 2018; Shi et al. 2023). The majority of measured NFG abundances, however, were one to two orders of magnitude higher than those reported in other studies on forest ecosystems dominated by ectomycorrhizal‐associated deciduous tree species (Shi et al. 2023; Ernfors et al. 2011; Singavarapu et al. 2023).

NFGs associated with nitrification (nxrb, AOA) and denitrification (nirK) were relatively abundant (regardless of the treatment), entailing a high potential for those processes to occur in the soil at our site. Concerning nitrification, both AOA and AOB had relatively high abundances, denoting a high potential nitrification rate at the site, with archaeal being more abundant than bacterial nitrifiers. The observed relative abundances of AOA and AOB in our experiment closely mirror those documented by Zhang et al. (2015) in an N manipulation experiment conducted in a subtropical deciduous forest, including both above and below‐canopy N additions, with similar doses considered at our site (25 and 50 kg N ha^−1^y^−1^). The authors reported a significant reduction in AOA and AOB abundances, particularly at higher application doses, following the above‐canopy fertilization approach. In contrast, the below‐canopy treatment reduced AOA abundances only at the higher dose. However, our results do not corroborate this observed reduction in nitrifier abundances, at least not 4 years after the experiment started. Within the NFGs involved in denitrification processes, the abundance of bacterial nirK was two orders of magnitude higher than that of fungal nirK, highlighting the potential limited contribution of the fungal community to denitrification at our experimental site. The relatively low abundance of N‐fixing bacteria NFG may indicate that the soil microbial community invested less energy in N‐fixing processes. Previous studies reported the preference of N‐fixing bacteria for soil with high C/N ratios, and their activity may diminish under elevated N atmospheric deposition (Levy‐Booth et al. 2014; Wallenstein et al. 2006; Hallin et al. 2009). Overall, the NFGs data suggested a relatively low potential for N fixation, but high potential rates for denitrification and nitrification. These findings hint that although 4 years of N fertilizations did not alter soil microbial functionality related to N processes, microbial N demand might already have been met. This could have led to reduced N retention, particularly in the N60 treatment, and to increased N losses from the forest ecosystem, such as gaseous N emissions and nitrate leaching (Niu et al. 2016; Levy‐Booth et al. 2014), as suggested by δ^15^N values, thus partially confirming our second hypothesis.

### 4.3 Do Tree Soil‐Tree N Pathways and N‐Uptake Strategies Change Between Above‐Canopy and Soil Fertilization?

The enrichment factor (EF) is often used as an integrated measure of N cycling processes in the soil (Emmett et al. 1998; Amundson et al. 2003). In the present study, the inability of EF to detect any differences among the fertilization treatments applied confirmed that the simple comparison of δ^15^N between plant foliage and soil N cannot always provide clear evidence of possible alteration in N plant–soil dynamics (Handley and Scrimgeour 1997; Robinson 2001). Instead, in our study, offsets between main forest compartments (Δδ^15^N) allowed us to ascertain how different treatments affected pathways related to soil–plant N interactions (Figure 1, Table 1).

Plant δ^15^N is the result of multiple factors, such as the sources of N (i.e., soil, atmospheric deposition, N_2_ fixation, N canopy uptake), the forms of soil N used, the influences of mycorrhizal associations, fractionations during and after N uptake by plants, and plant phenology (Högberg 1997; Craine et al. 2015). If those processes had a similar influence, no matter the fertilization approach, we should have observed the same N dynamics in above‐canopy vs. direct‐to‐soil fertilizations, as reflected with similar Δδ^15^N and relationships across compartments, with particular reference to δ^15^N in ERT, roots, and leaves. Yet, this was not the case in our study. Both N30 and N60 resulted in an elevated N concentration and more positive δ^15^N values in the ERT as well as in the soil compared to N0. The higher N concentration in the ERT suggests a potential role of ectomycorrhizal fungi in the immobilization of N derived from direct‐to‐soil fertilization (either directly or indirectly, by taking up more ^15^N as a result of possible N losses). Moreover, the lack of a concurrent increase in N concentration in fine roots and foliar tissues suggests that the excess of N immobilized in the ERT compartment may not be proportionally transferred to the host plants. The mechanism by which ectomycorrhizal fungi balance N availability between transferring N to the host plant and meeting their own N demand remains unclear. Näsholm et al. (2013) hypothesized that N addition to the soil should override the N demand of mycorrhizal fungi, reducing the proportion of N they immobilize and thereby increasing the supply of N to the tree canopy. However, the N concentration and isotopic composition do not appear to fully support this hypothesis. This suggests that fertilization may have altered the dependence of trees on mycorrhizal association as well as the N source of ectomycorrhizal fungi, that is, shifting from organic to inorganic (Pellitier et al. 2021), the latter being more readily available through the fertilization. Indeed, the different Δδ^15^N values observed from soil to ERT and from Litter OH to ERT in N60 compared to N0 may support the hypothesis of a potential change in the N source of ectomycorrhizal fungi.

By contrast, the N30A treatment showed low δ^15^N values in the below‐ground organic compartments, with statistically significant differences observed only in fine roots compared to the N0. The ^15^N depletion in fine roots observed in N30A compared to the N0 could be explained by increased enzymatic activities of ectomycorrhizal fungi, enabling host plants to access N from fresh, litter‐derived sources that are less enriched in ^15^N (Hobbie 2008). Alternatively, it has been proposed that ectomycorrhizal fungi preferentially retain ^15^N‐enriched N and transfer N depleted in ^15^N to their host plants (Ouimette et al. 2013; Högberg 1997), which is then reflected in more negative δ^15^N values for trees with ectomycorrhizal associations than for non‐mycorrhizal trees (Craine et al. 2009). In both scenarios, the depletion of ^15^N in fine root tissues in N30A points to a strong dependency of plants on ectomycorrhizal fungi activity for N uptake, and hence a higher N retention in the system.

The ^15^N depletion observed in the decomposed litter (OF layer) in N30A compared to the N30, coincided with a higher N concentration, which cannot be explained by N enrichment in partially decomposed litter (OF), as no significant differences were observed among all treatments for N content. These findings suggest that the above‐canopy fertilization may affect litter quality (e.g., increase in lignin to cellulose ratio) with a likely inhibitory effect on litter decomposition (Morrison et al. 2018). Indeed, the negative effect of added N on decomposition rates has been extensively documented in the literature (Knorr et al. 2005; Janssens and Luyssaert 2009), particularly in high‐lignin litter types, which is generally the case in mature beech forest (Sariyildiz and Anderson 2003; Trap et al. 2013). This effect is often attributed to a reduction in microbial extracellular enzyme activity, which may respond adversely to N addition (Magill and Aber 1998; Sinsabaugh et al. 2002; Knorr et al. 2005; Morrison et al. 2018).

Leaves were consistently enriched in ^15^N compared to fine root tissues, with greater responses in the N30A compared to the N0 and direct‐to‐soil treatments. Evans et al. (1996) reported that this enrichment pattern is common in plants utilizing NO_3_^−^ as their N source (such as beech trees, see Templer and Dawson 2004; Leberecht et al. 2016), which is attributed to the NO_3_^−^ reduction processes occurring during plant assimilation and translocation. However, the distinct higher Δδ^15^N_(fine roots‐leaves)_ in leaves observed in the N30A compared to N0 may be attributed to a potential foliar N uptake, facilitated by the above‐canopy fertilization treatment. Furthermore, the Δδ^15^N between fresh leaves and litter OF revealed that both above canopy and direct‐to‐soil treatments caused a change in the N resorption mechanism prior to leaf shading. Indeed, while litter OF appeared enriched in ^15^N compared to the fresh leaves in N0, the opposite pattern was observed in all treatments, regardless of the fertilization approach.

## 5 Conclusions

This study provides new insights into how different nitrogen fertilization treatments, used to simulate an increase in atmospheric nitrogen deposition, influence forest nitrogen dynamics across multiple ecosystem compartments. By integrating isotopic, chemical, and microbial indicators, we offer a more comprehensive understanding of divergence in ecosystem N pathways under above‐canopy and soil fertilization.

Although the use of natural ^15^N abundances may pose challenges in quantifying forest ecosystem N retention vs. losses under different fertilization approaches, our results indicate that δ^15^N is a more reliable and sensitive indicator of changes in ecosystem N dynamics, particularly in distinguishing the effects of above‐canopy vs. soil fertilizations, compared to N concentrations alone. The δ^15^N values measured in forest compartments under the soil N treatments (particularly those receiving the highest dose, N60) revealed an incipient alteration of the N cycle, which was, however, not observed under the above‐canopy treatment (N30A). Yet N concentrations were minimally affected by the treatments. Following 4 years of experimental nitrogen additions to an already N‐rich forest ecosystem, we found limited evidence of N saturation, at least in the short term. This contrasts with earlier observations from soil fertilization experiments using higher doses in forest ecosystems (Magill et al. 2000; Ochoa‐Hueso et al. 2013). Therefore, we urge the next generation of N manipulation experiments to more realistically simulate increases in N deposition (both in terms of doses applied, methods, and, most importantly, duration, Högberg et al. 2024) to account for homeostatic adjustments of the ecosystems and to follow any development in the ecosystem N saturation conditions and changes in tree N uptake strategies.

## Author Contributions

**Alessandra Teglia:** conceptualization, data curation, formal analysis, investigation, visualization, writing – original draft, writing – review and editing. **Cristiana Sbrana:** data curation, methodology, writing – review and editing. **Stefania Mattana:** data curation, methodology, writing – review and editing. **Andrea Scartazza:** formal analysis, methodology, writing – review and editing. **Matteo Bucci:** data curation, writing – review and editing. **Paola Gioacchini:** formal analysis, writing – review and editing. **Graziella Marcolini:** data curation, writing – review and editing. **Enrico Muzzi:** formal analysis, writing – review and editing. **Dario Ravaioli:** investigation, writing – review and editing. **Angela Ribas:** formal analysis, resources, writing – review and editing. **Federico Magnani:** funding acquisition, methodology, writing – review and editing. **Rossella Guerrieri:** conceptualization, methodology, supervision, writing – original draft, writing – review and editing.

## Conflicts of Interest

The authors declare no conflicts of interest.

## Supporting information


**Table S1:** Sequence primers, annaeling temperature, bp of each target gene.

## Data Availability

Data are available from the Dryad Digital Repository: https://doi.org/10.5061/dryad.3r2280gw1 (Teglia et al. 2025).

## References

[gcb70534-bib-0001] Aber, J. D. 2002. “Nitrogen Saturation in Temperate Forest Ecosystems: Current Theory, Remaining Questions and Recent Advances.” In Progress in Plant Nutrition: Plenary Lectures of the XIV International Plant Nutrition Colloquium, 179–188. Springer. 10.1007/978-94-017-2789-1_13.

[gcb70534-bib-0002] Aber, J. D. , W. McDowell , K. Nadelhoffer , et al. 1998. “Nitrogen Saturation in Temperate Forest Ecosystems.” Bioscience 48, no. 11: 921–934. 10.2307/1313296.

[gcb70534-bib-0003] Amundson, R. , A. Austin , E. Schuur , et al. 2003. “Global Patterns of the Isotopic Composition of Soil and Plant Nitrogen.” Global Biogeochemical Cycles 17, no. 1: 6‐1–6‐14. 10.1029/2001GB001807.

[gcb70534-bib-0004] Arnhold, E. 2013. “Package in the R Environment for Analysis of Variance and Complementary Analyses.” Brazilian Journal of Veterinary Research and Animal Science 50, no. 6: 488–492.

[gcb70534-bib-0005] Bebber, D. P. 2021. “The Gap Between Atmospheric Nitrogen Deposition Experiments and Reality.” Science of the Total Environment 801: 149774. 10.1016/j.scitotenv.2021.149774. 34470727

[gcb70534-bib-0006] Billow, C. , P. Matson , and B. Yoder . 1994. “Seasonal Biochemical‐Changes in Coniferous Forest Canopies and Their Response to Fertilization.” Tree Physiology 14: 563–574. 14967674 10.1093/treephys/14.6.563

[gcb70534-bib-0008] Bortolazzi, A. , L. Da Ros , M. Rodeghiero , R. Tognetti , G. Tonon , and M. Ventura . 2021. “The Canopy Layer, a Biogeochemical Actor in the Forest N‐Cycle.” Science of the Total Environment 776, no. 146: 146024. 10.1016/j.scitotenv.2021.146024.

[gcb70534-bib-0009] Cecchini, G. , A. Andreetta , A. Marchetto , and S. Carnicelli . 2021. “Soil Solution Fluxes and Composition Trends Reveal Risks of Nitrate Leaching From Forest Soils of Italy.” Catena 200, no. 105: 105175. 10.1016/j.catena.2021.105175.

[gcb70534-bib-0010] Craine, J. M. , E. N. J. Brookshire , M. D. Cramer , et al. 2015. “Ecological Interpretations of Nitrogen Isotope Ratios of Terrestrial Plants and Soils.” Plant and Soil 396, no. 1: 1–26. 10.1007/s11104-015-2542-1.

[gcb70534-bib-0011] Craine, J. M. , A. J. Elmore , M. P. M. Aidar , et al. 2009. “Global Patterns of Foliar Nitrogen Isotopes and Their Relationships With Climate, Mycorrhizal Fungi, Foliar Nutrient Concentration, and Nitrogen Availability.” New Phytologist 183, no. 4: 980–992. 10.1111/j.1469-8137.2009.02917.x. 19563444

[gcb70534-bib-0012] Craine, J. M. , A. J. Elmore , L. Wang , et al. 2018. “Isotopic Evidence for Oligotrophication of Terrestrial Ecosystems.” Nature Ecology & Evolution 2: 1735–1744. 10.1038/s41559-018-0694-0. 30349095

[gcb70534-bib-0013] Da Ros, L. , M. Rodeghiero , C. L. Goodale , et al. 2023. “Canopy 15 N Fertilization Increases Short‐Term Plant N Retention Compared to Ground Fertilization in an Oak Forest.” Forest Ecology and Management 539, no. 121: 121001. 10.1016/j.foreco.2023.121001.

[gcb70534-bib-0014] Dail, D. , D. Y. Hollinger , E. A. Davidson , et al. 2009. “Distribution of Nitrogen‐15 Tracers Applied to the Canopy of a Mature Spruce‐Hemlock Stand, Howland, Maine, USA.” Oecologia 160: 589–599. 10.1007/s00442-009-1325-x. 19352716

[gcb70534-bib-0015] de Mendiburu, F. , and M. Yaseen . 2020. Agricolae: Statistical Procedures for Agricultural Research (Version 1.4.0) [R Package]. https://cran.r‐Project.org/package=agricolae.

[gcb70534-bib-0016] de Schrijver, A. , P. de Frenne , E. Ampoorter , et al. 2011. “Cumulative Nitrogen Input Drives Species Loss in Terrestrial Ecosystems.” Global Ecology and Biogeography 20: 803–816. 10.1111/j.1466-8238.2011.00652.x.

[gcb70534-bib-0017] Du, E. , and W. de Vries . 2024. “Impacts of Nitrogen Deposition on Forest Productivity and Carbon Sequestration.” In Atmospheric Nitrogen Deposition to Global Forests, edited by E. Du and W. de Vries , 59–76. Academic Press. 10.1016/B978-0-323-91140-5.00016-6.

[gcb70534-bib-0018] Du, E. , and W. de Vries . 2025. “Links Between Nitrogen Limitation and Saturation in Terrestrial Ecosystems.” Global Change Biology 31: e70271. 10.1111/gcb.70271. 40444577

[gcb70534-bib-0019] Emmett, B. A. , O. J. Kjonaas , P. Gundersen , C. Koopmans , A. Tietema , and D. Sleep . 1998. “Natural Abundance of ^15^N in Forests Across a Nitrogen Deposition Gradient.” Forest Ecology and Management 101, no. 1–3: 9–18. 10.1016/S0378-1127(97)00126-5.

[gcb70534-bib-0020] Ernfors, M. , T. Rutting , and L. Klemedtsson . 2011. “Increased Nitrous Oxide Emissions From a Drained Organic Forest Soil After Exclusion of Ectomycorrhizal Mycelia.” Plant and Soil 343: 161–170. 10.1007/s11104-010-0709-3.

[gcb70534-bib-0021] Etzold, S. , M. Ferretti , G. J. Reinds , et al. 2020. “Nitrogen Deposition Is the Most Important Environmental Driver of Growth of Pure, Even‐Aged and Managed European Forests.” Forest Ecology and Management 458, no. 117: 117762. 10.1016/j.foreco.2019.117762.

[gcb70534-bib-0022] Evans, R. D. , A. J. Bloom , S. S. Sukrapanna , and J. R. Ehleringer . 1996. “Nitrogen Isotope Composition of Tomato ( *Lycopersicon esculentum* Mill. cv. T‐5) Grown Under Ammonium or Nitrate Nutrition.” Plant, Cell & Environment 19, no. 11: 1317–1323. 10.1111/j.1365-3040.1996.tb00010.x.

[gcb70534-bib-0023] Ferraretto, D. , R. Nair , N. W. Shah , et al. 2022. “Forest Canopy Nitrogen Uptake Can Supply Entire Foliar Demand.” Functional Ecology 36, no. 5: 933–949. 10.1111/1365-2435.14005.

[gcb70534-bib-0024] Fry, B. 2006. Stable Isotope Ecology, 21–39. Springer. 10.1007/0-387-33745-8.

[gcb70534-bib-0025] Gaige, E. , D. B. Dail , D. Y. Hollinger , et al. 2007. “Changes in Canopy Processes Following Whole‐Forest Canopy Nitrogen Fertilization of a Mature Spruce‐Hemlock Forest.” Ecosystems 10, no. 7: 1133–1147. 10.1007/s10021-007-9081-4.

[gcb70534-bib-0026] Galloway, J. N. , F. J. Dentener , D. G. Capone , et al. 2004. “Nitrogen Cycles: Past, Present, and Future.” Biogeochemistry 70: 153–226. 10.1007/s10533-004-0370-0.

[gcb70534-bib-0027] Galloway, J. N. , A. R. Townsend , J. W. Erisman , et al. 2008. “Transformation of the Nitrogen Cycle: Recent Trends, Questions, and Potential Solutions.” Science 320, no. 5878: 889–892. 10.1126/science.1136674. 18487183

[gcb70534-bib-0028] Gebauer, G. , and E. D. Schulze . 1991. “Carbon and Nitrogen Isotope Ratios in Different Compartments of a Healthy and a Declining *Picea abies* Forest in the Wchtelgebirge, Northeastern Bavaria (Germany).” Oecologia 87: 198–207. 10.1007/BF00325258. 28313836

[gcb70534-bib-0029] Guerrieri, R. , J. Cáliz , S. Mattana , et al. 2024. “Substantial Contribution of Tree Canopy Nitrifiers to Nitrogen Fluxes in European Forests.” Nature Geoscience 17: 130–136. 10.1038/s41561-023-01364-3.

[gcb70534-bib-0030] Guerrieri, R. , P. Templer , and F. Magnani . 2021. “Canopy Exchange and Modification of Nitrogen Fluxes in Forest Ecosystems.” Current Forestry Reports 7: 115–137. 10.1007/s40725-021-00141-y.

[gcb70534-bib-0031] Gundersen, P. , I. K. Schmidt , and K. Raulund‐Rasmussen . 2006. “Leaching of Nitrate From Temperate Forests: Effects of Air Pollution and Forest Management.” Environmental Reviews 14, no. 1: 1–57. 10.1139/a05-015.

[gcb70534-bib-0032] Hallin, S. , C. M. Jones , M. Schloter , and L. Philippot . 2009. “Relationship Between N‐Cycling Communities and Ecosystem Functioning in a 50‐Year‐Old Fertilization Experiment.” ISME Journal 3: 597–605. 10.1038/ismej.2008.128. 19148144

[gcb70534-bib-0033] Han, Q. , D. Kabeya , and Y. Inagaki . 2017. “Influence of Reproduction on Nitrogen Uptake and Allocation to New Organs in *Fagus Crenata* .” Tree Physiology 37, no. 10: 1436–1443. 10.1093/treephys/tpx083. 28985424

[gcb70534-bib-0034] Handley, L. L. , and C. M. Scrimgeour . 1997. “Terrestrial Plant Ecology and 15 N Natural Abundance.” Advances in Ecological Research 27: 133–212. 10.1016/S0065-2504(08)60073-2.

[gcb70534-bib-0035] Hobbie, E. , and A. Ouimette . 2009. “Controls of Nitrogen Isotope Patterns in Soil Profiles.” Biogeochemistry 95, no. 2: 355–371. 10.1007/s10533-009-9328-6.

[gcb70534-bib-0036] Hobbie, E. A. , and J. V. Colpaert . 2003. “Nitrogen Availability and Colonization by Mycorrhizal Fungi Correlate With Nitrogen Isotope Patterns in Plants.” New Phytologist 157: 115–126. 10.1046/j.1469-8137.2003.00657.x. 33873704

[gcb70534-bib-0038] Hobbie, S. E. 2008. “Nitrogen Effects on Litter Decomposition: A Five‐Year Experiment in Eight Temperate Grassland and Forest Sites.” Ecology 89, no. 9: 2633–2644. 10.1890/07-1119.1. 18831184

[gcb70534-bib-0039] Högberg, P. 1990. “Forests Losing Large Quantities of Nitrogen Have Elevated Nitrogen 15 N/14 N Ratios.” Oecologia 84: 229–231. 10.1007/BF00318276. 28312757

[gcb70534-bib-0040] Högberg, P. 1997. “15 N Natural Abundance in Soil–Plant Systems.” New Phytologist 137: 179–203. 10.1046/j.1469-8137.1997.00808.x. 33863175

[gcb70534-bib-0041] Högberg, P. , R. W. Lucas , M. N. Högberg , et al. 2024. “What Happens to Trees and Soils During Five Decades of Experimental Nitrogen Loading?” Forest Ecology and Management 553, no. 121: 121644. 10.1016/j.foreco.2023.121644.

[gcb70534-bib-0042] Hyvönen, R. , T. Persson , S. Andersson , B. Olsson , G. I. Ågren , and S. Linder . 2008. “Impact of Long Term Nitrogen Addition on Carbon Stocks in Trees and Soils in Northern Europe.” Biogeochemistry 89: 121–137. 10.1007/s10533-007-9121-3.

[gcb70534-bib-0043] Janssens, I. A. , and S. Luyssaert . 2009. “Carbon Cycle: Nitrogen's Carbon Bonus.” Nature Geoscience 2: 318–319. 10.1038/ngeo505.

[gcb70534-bib-0044] Kaakinen, S. , R. Piispanen , S. Lehto , et al. 2009. “Growth, Wood Chemistry, and Fibre Length of Norway Spruce in a Long Term Nutrient Optimization Experiment.” Canadian Journal of Forest Research 39: 410–419. 10.1139/X08-180.

[gcb70534-bib-0045] Kendall, C. , E. M. Elliott , and S. D. Wankel . 2007. “Tracing Anthropogenic Inputs of Nitrogen to Ecosystems.” In Stable Isotopes in Ecology and Environmental Science, edited by R. Michener and K. Lajtha , 375–449. Blackwell Publishing.

[gcb70534-bib-0046] Knorr, M. , S. D. Frey , and P. S. Curtis . 2005. “Nitrogen Additions and Litter Decomposition: A Meta‐Analysis.” Ecology 86, no. 12: 3252–3257. 10.1890/05-0150.

[gcb70534-bib-0047] Komsta, L. 2011. Outliers: Tests for Outliers (Version 0.14) [R Package]. https://CRAN.R‐project.org/package=outliers.

[gcb70534-bib-0048] Leberecht, M. , M. Dannenmann , J. Tejedor , J. Simon , H. Rennenberg , and A. Polle . 2016. “Segregation of Nitrogen Use Between Ammonium and Nitrate of Ectomycorrhizas and Beech Trees.” Plant, Cell & Environment 39, no. 12: 2691–2700. 10.1111/pce.12820. 27569258

[gcb70534-bib-0049] Levy‐Booth, D. , C. Prescott , and S. Grayston . 2014. “Microbial Functional Genes Involved in Nitrogen Fixation, Nitrification, and Denitrification in Forest.” Soil Biology and Biochemistry 75: 11–25. 10.1016/j.soilbio.2014.03.021.

[gcb70534-bib-0050] Liu, Y. , X. Tan , S. Fu , and W. Shen . 2022. “Canopy and Understory Nitrogen Addition Alters Organic Soil Bacterial Communities but Not Fungal Communities in a Temperate Forest.” Frontiers in Microbiology 13, no. 888: 888121. 10.3389/fmicb.2022.888121. 35756006 PMC9226683

[gcb70534-bib-0051] Magill, A. , J. Aber , G. Berntson , et al. 2000. “Long‐Term Nitrogen Additions and Nitrogen Saturation in Two Temperate Forests.” Ecosystems 3: 238–253. 10.1007/s100210000023.

[gcb70534-bib-0052] Magill, A. H. , and J. D. Aber . 1998. “Long‐Term Effects of Experimental Nitrogen Additions on Foliar Litter Decay and Humus Formation in Forest Ecosystems.” Plant and Soil 203: 301–311.

[gcb70534-bib-0053] Magill, A. H. , J. D. Aber , W. S. Currie , et al. 2004. “Ecosystem Response to 15 Years of Chronic Nitrogen Additions at the Harvard Forest LTER, Massachusetts, USA.” Forest Ecology and Management 196, no. 1: 7–28. 10.1016/j.foreco.2004.03.033.

[gcb70534-bib-0054] Magnani, F. , M. Mencuccini , M. Borghetti , et al. 2007. “The Human Footprint in the Carbon Cycle of Temperate and Boreal Forests.” Nature 447, no. 7146: 848–850. 10.1038/nature05847. 17568744

[gcb70534-bib-0055] Marchetto, A. , S. Arisci , G. A. Tartari , R. Balestrini , and D. Tait . 2014. “Stato ed. Evoluzione Temporale Della Composizione Chimica Delle Deposizioni Atmosferiche Nelle Aree Forestali Della rete CONECOFOR.” Forest@‐Journal of Silviculture and Forest Ecology 11, no. 1: 72–85. 10.3832/efor1003-011.

[gcb70534-bib-0056] Mason, R. E. , J. M. Craine , N. K. Lany , et al. 2022. “Evidence, Causes, and Consequences of Declining Nitrogen Availability in Terrestrial Ecosystems.” Science 376, no. 6590: eabh3767. 10.1126/science.abh3767. 35420945

[gcb70534-bib-0057] Meints, V. W. , L. V. Boone , and L. T. Kurtz . 1975. “Natural 15 N Abundance in Soil, Leaves, and Grain as Influenced by Long‐Term Additions of Fertilizer N at Several Rates.” Journal of Environmental Quality 4, no. 4: 486–490. 10.2134/jeq1975.00472425000400040024x.

[gcb70534-bib-0058] Morrison, E. W. , A. Pringle , L. T. van Diepen , and S. D. Frey . 2018. “Simulated Nitrogen Deposition Favors Stress‐Tolerant Fungi With Low Potential for Decomposition.” Soil Biology and Biochemistry 125: 75–85. 10.1016/j.soilbio.2018.07.003.

[gcb70534-bib-0059] Nadelhoffer, K. J. , B. A. Emmett , P. Gundersen , et al. 1999. “Nitrogen Deposition Makes a Minor Contribution to Carbon Sequestration in Temperate Forests.” Nature 398: 145–148. 10.1038/18205.

[gcb70534-bib-0060] Näsholm, T. , P. Högberg , O. Franklin , et al. 2013. “Are Ectomycorrhizal Fungi Alleviating or Aggravating Nitrogen Limitation of Tree Growth in Boreal Forests?” New Phytologist 198, no. 1: 214–221. 10.1111/nph.12139. 23356503

[gcb70534-bib-0061] Niu, S. , A. Classen , J. S. Dukes , et al. 2016. “Global Patterns and Substrate‐Based Mechanisms of the Terrestrial Nitrogen Cycle.” Ecology Letters 19, no. 6: 697–709. 26932540 10.1111/ele.12591

[gcb70534-bib-0063] Ochoa‐Hueso, R. , F. T. Maestre , A. de los Ríos , et al. 2013. “Nitrogen Deposition Alters Nitrogen Cycling and Reduces Soil Carbon Content in Low‐Productivity Semiarid Mediterranean Ecosystems.” Environmental Pollution 179: 185–193. 23685631 10.1016/j.envpol.2013.03.060 PMC4427509

[gcb70534-bib-0064] Ouimette, A. , D. Guo , E. Hobbie , et al. 2013. “Insights Into Root Growth, Function, and Mycorrhizal Abundance From Chemical and Isotopic Data Across Root Orders.” Plant and Soil 367: 313–326. 10.1007/s11104-012-1464-4.

[gcb70534-bib-0065] Oulehle, F. , P. Šamonil , O. Urban , et al. 2025. “Growth and Assemblage Dynamics of Temperate Forest Tree Species Match Physiological Resilience to Changes in Atmospheric Chemistry.” Global Change Biology 31, no. 3: e70147. 10.1111/gcb.70147. 40135407 PMC11938019

[gcb70534-bib-0066] Pellitier, P. T. , D. R. Zak , W. A. Argiroff , and R. A. Upchurch . 2021. “Coupled Shifts in Ectomycorrhizal Communities and Plant Uptake of Organic Nitrogen Along a Soil Gradient: An Isotopic Perspective.” Ecosystems 24: 1976–1990. 10.1007/s10021-021-00628-6.

[gcb70534-bib-0067] Peterson, B. J. , and B. Fry . 1987. “Stable Isotopes in Ecosystem Studies.” Annual Review of Ecology and Systematics 18: 293–320.

[gcb70534-bib-0068] R Core Team . 2017. R: A language and Environment for Statistical Computing (Version 3.4.0) [Software]. R Foundation for Statistical Computing. https://www.R‐project.org/.

[gcb70534-bib-0069] Rennenberg, H. , and M. Dannenmann . 2015. “Nitrogen Nutrition of Trees in Temperate Forests—The Significance of Nitrogen Availability in the Pedosphere and Atmosphere.” Forests 6, no. 8: 2820–2835. 10.3390/f6082820.

[gcb70534-bib-0070] Robinson, D. 2001. “Delta N‐15 as an Integrator of the Nitrogen Cycle.” Trends in Ecology & Evolution 16, no. 3: 153–162. 10.1016/s0169-5347(00)02098-x. 11179580

[gcb70534-bib-0071] Sariyildiz, T. , and J. M. Anderson . 2003. “Decomposition of Sun and Shade Leaves From Three Deciduous Tree Species, as Affected by Their Chemical Composition.” Biology and Fertility of Soils 37, no. 2: 137–146. 10.1007/s00374-002-0569-y.

[gcb70534-bib-0072] Scartazza, A. , C. Sbrana , E. D'Andrea , G. Matteucci , N. Rezaie , and M. Lauteri . 2023. “Above‐ and Belowground Interplay: Canopy CO_2_ Uptake, Carbon and Nitrogen Allocation and Isotope Fractionation Along the Plant Ectomycorrhiza Continuum.” Plant, Cell & Environment 46, no. 3: 889–900. 10.1111/pce.14519. 36541420

[gcb70534-bib-0073] Schulte‐Uebbing, L. , and W. de Vries . 2018. “Global‐Scale Impacts of Nitrogen Deposition on Tree Carbon Sequestration in Tropical, Temperate, and Boreal Forests: A Meta‐Analysis.” Global Change Biology 24, no. 2: e416–e431. 10.1111/gcb.13862. 29034987

[gcb70534-bib-0074] Shi, X. , L. Wang , J. Sun , M. Lucas‐Borja , and J. Wang . 2023. “Nitrogen Cycling‐Related Functional Genes Exhibit Higher Sensibility in Soil Than Leaf Phyllosphere of Different Tree Species in the Subtropical Forests.” Plant and Soil 493, no. 1–2: 173–185.

[gcb70534-bib-0075] Sievering, H. , T. Tomasewski , and J. Torizzo . 2007. “Canopy Uptake of Atmospheric N Deposition at a Conifer Forest. Part I. Canopy N Budget, Photosynthetic Efficiency and Net Ecosystem Exchange.” Tellus B 59, no. 6: 483–492.

[gcb70534-bib-0076] Singavarapu, B. , J. Du , R. Beugnon , et al. 2023. “Functional Potential of Soil Microbial Communities and Their Subcommunities Varies With Tree Mycorrhizal Type and Tree Diversity.” Microbiology Spectrum 11, no. 2: 04578. 10.1128/spectrum.04578-22. PMC10111882 36951585

[gcb70534-bib-0077] Sinsabaugh, R. L. , M. M. Carreiro , and D. A. Repert . 2002. “Allocation of Extracellular Enzymatic Activity in Relation to Litter Composition, N Deposition, and Mass Loss.” Biogeochemistry 60, no. 1: 1–24.

[gcb70534-bib-0078] Sparks, J. P. 2009. “Ecological Ramifications of the Direct Foliar Uptake of Nitrogen.” Oecologia 159, no. 1: 1–13. 10.1007/s00442-008-1188-6. 18975011

[gcb70534-bib-0079] Steffen, W. , K. Richardson , J. Rockström , et al. 2015. “Planetary Boundaries: Guiding Human Development on a Changing Planet.” Science 347, no. 6223: 1,259,855. 10.1126/science.1259855. 25592418

[gcb70534-bib-0080] Takahashi, M. , Z. Feng , T. A. Mikhailova , et al. 2020. “Air Pollution Monitoring and Tree and Forest Decline in East Asia: A Review.” Science of the Total Environment 742, no. 140: 140288. 10.1016/j.scitotenv.2020.140288. 32721711

[gcb70534-bib-0081] Tang, Y. , G. Yu , X. Zhang , Q. Wang , J. Ge , and S. Liu . 2018. “Changes in Nitrogen‐Cycling Microbial Communities With Depth in Temperate and Subtropical Forest Soils.” Applied Soil Ecology 124: 218–228.

[gcb70534-bib-0082] Teglia, A. , D. Di Baccio , G. Matteucci , et al. 2022. “Effects of Simulated Nitrogen Deposition on the Nutritional and Physiological Status of Beech Forests at Two Climatic Contrasting Sites in Italy.” Science of the Total Environment 834, no. 155: 155362. 10.1016/j.scitotenv.2022.155362. 35460784

[gcb70534-bib-0083] Teglia, A. , S. Mattana , and R. Guerrieri . 2025. “Data from: Forest Nitrogen Dynamics in Response to Increasing Nitrogen Deposition: Comparing Above‐Canopy and Soil Fertilizations in a Mature Beech Forest”. 10.5061/dryad.3r2280gw1. PMC12506859 41060225

[gcb70534-bib-0084] Templer, P. H. , M. A. Arthur , G. M. Lovett , and K. C. Weathers . 2007. “Plant and Soil Natural Abundance δ15N: Indicators of Relative Rates of Nitrogen Cycling in Temperate Forest Ecosystems.” Oecologia 153, no. 2: 399–406. 17479293 10.1007/s00442-007-0746-7

[gcb70534-bib-0085] Templer, P. H. , and T. E. Dawson . 2004. “Nitrogen Uptake by Four Tree Species of the Catskill Mountains, New York: Implications for Nitrogen Cycling.” Plant and Soil 262, no. 1: 251–261.

[gcb70534-bib-0086] Terrer, C. , R. B. Jackson , I. C. Prentice , et al. 2019. “Nitrogen and Phosphorus Constrain the CO_2_ Fertilization of Global Plant Biomass.” Nature Climate Change 9, no. 7: 684–689.

[gcb70534-bib-0087] Trap, J. , S. Hättenschwiler , I. Gattin , and M. Aubert . 2013. “Forest Ageing: An Unexpected Driver of Beech Leaf Litter Quality Variability in European Forests With Strong Consequences on Soil Processes.” Forest Ecology and Management 302: 338–345.

[gcb70534-bib-0088] Wallenstein, M. D. , W. T. Peterjohn , and W. H. Schlesinger . 2006. “N Fertilization Effects on Denitrification and N Cycling in an Aggrading Forest.” Ecological Applications 16, no. 6: 2168–2176. 17205895 10.1890/1051-0761(2006)016[2168:nfeoda]2.0.co;2

[gcb70534-bib-0089] Wang, R. , D. Goll , Y. Balkanski , et al. 2017. “Global Forest Carbon Uptake due to Nitrogen and Phosphorus Deposition From 1850 to 2100.” Global Change Biology 23, no. 12: 4854–4872. 10.1111/gcb.13727. 28513916

[gcb70534-bib-0090] Wang, S. , Y. Zhang , W. Ju , et al. 2020. “Recent Global Decline of CO_2_ Fertilization Effects on Vegetation Photosynthesis.” Science 370, no. 6522: 1295–1300. 10.1126/science.abb7772. 33303610

[gcb70534-bib-0091] Wickham, H. 2016. Ggplot2: Elegant Graphics for Data Analysis. Springer‐Verlag. https://ggplot2.tidyverse.org.

[gcb70534-bib-0092] Wickham, H. , M. Averick , J. Bryan , et al. 2019. “Welcome to the Tidyverse.” Journal of Open Source Software 4, no. 43: 01686. 10.21105/joss.01686.

[gcb70534-bib-0093] Yoneyama, T. 1996. “Characterization of Natural 15 N Abundance of Soils.” In Mass Spectrometry of Soils, edited by T. W. Boutton and S. Yamasaki , 26. M. Dekker.

[gcb70534-bib-0094] Yves, R. 2012. “Lavaan: An R Package for Structural Equation Modeling.” Journal of Statistical Software 48, no. 2: 1–36. 10.18637/jss.v048.i02.

[gcb70534-bib-0095] Zaehle, S. 2013. “Terrestrial Nitrogen‐Carbon Cycle Interactions at the Global Scale.” Philosophical Transactions of the Royal Society, B: Biological Sciences 368, no. 1621: 20,130,125. 10.1098/rstb.2013.0125. PMC3682745 23713123

[gcb70534-bib-0096] Zhang, W. , W. Shen , S. Zhu , et al. 2015. “Can Canopy Addition of Nitrogen Better Illustrate the Effect of Atmospheric Nitrogen Deposition on Forest Ecosystem?” Scientific Reports 5, no. 11: 11245. 10.1038/srep11245. 26059183 PMC4462050

