# Supplementary material for: Forest Nitrogen Dynamics in Response to Increasing Nitrogen Deposition: Comparing Above‐Canopy and Soil Fertilizations in a Mature Beech Forest
Source: Glob Chang Biol. 2025 Oct 8;31(10):e70534. doi: 10.1111/gcb.70534 (PMC12506859; doi:10.1111/gcb.70534)
Supplement: Supplementary file 1 — Table S1: Sequence primers, annaeling temperature, bp of each target gene. [file GCB-31-e70534-s001.docx]

## Supplementary materials

### Quantitative PCR analyses

Single PCR reactions were prepared in a total volume of 10 μl containing the following: 5 μl of KAPA SYBR® FAST qPCR Master Mix (Roche), 0.25 μl of forward and reverse primer (10 μM) (Metabion); 0.25 μl of dimetyl sulfoxide, DMSO, (Sigma); 1,5 μl H2O, and 2.5 μl template DNA (4 ng μl^−1^). qPCR was performed by using LightCycler 480 (Roche). At the end of each run, melting curve analysis of the PCR products was conducted to confirm that the fluorescence signal came from specific PCR products and not from primer-dimers or other artifacts. Additionally, an agarose gel (2%) was run to check the correct size of amplicons.

The qPCR standards were obtained from the following sources: soil gDNA (bacterial *amoA*, *qnorB* and *nirk*Fungi); environmental clone N3-16 [AB62272] (archeal *amoA*); *Acidithiobacillus ferrooxidans* (*nifH*); *Nitrospira* sp. (*nxrB*); *Sinorhizobium melioti 1021* (*nirK* and *nosZ*) and *Ralstonia eutropha H16* (*nirS*). The PCR amplified DNA from the soil samples and the cultured microorganisms were purified using mi-Gel Extraction Kit (Metabion, Germany) and then standard curves were generated based on quantified PCR products with a series of 1:10 dilutions (R^2^ ≥ 0.99 for each gene). All the samples and standards were analyzed in triplicate and several negative controls were included. Amplification efficiencies were calculated as E = [10(−1/slope) -1]*100, with the following results: *nifH* 93% ; bacterial *amoA* 95%; archeal *amoA* 93%; *nxrB* 96% ;*nirK* 98%; *nosZ* 92%; *nirS* 89% ; *qnorB* 92% and fungal *nirK* 94%.

**Table S1:** Sequence primers, annaeling temperature, base pair (bp) of each target gene

| Target gene | Primers | Sequence | Annealig temperature (ºC) | Gene bp | Reference |
| --- | --- | --- | --- | --- | --- |
| nifH | nifHF | AAAGGYGGWATCGGYAARTCCACCAC | 55 | 458 | Rosh et al 2002 |
|  | nifHR | TTGTTSGCSGCRTACATSGCCATCAT |  |  |  |
| Archeal amoA | amo19F | ATGGTCTGGCTWAGACG | 55 | 624 | Leininger et al 2006 |
|  | amo643R | TCCCACTTWGACCARGCGGCCATCCA |  |  | Treusch et al 2005 |
| Bacterial amoA | amoA1F | GGGGTTTCTACTGGTGGT | 60 | 500 | Rotthauwe et al 1997 |
|  | amoA1R | CCCCTCKGSAAAGCCTTCTTC |  |  |  |
| Nitrobacter nxrB | nxrB1F | ACGTGGAGACCAAGCCGGG | 58.5 | 411 | Vanparys et al 2007 |
|  | nxrB1R | CCGTGCTGTTGAYCTCGTTGA |  |  |  |
| nirK | nirK876C | ATYGGCGGVCAYGGCGA | 58.5 | 164 | Harter et al 2014 |
|  | nirK1040 | GCCTCGATCAGRTTRTGGTT |  |  |  |
| nirS | nirScd3aF | AAC GYS AAG GAR ACS GG | 57 | 413 | Michotey et al (2000) |
|  | nirSR3cd | GAS TTC GGR TGS GTC TTS AYG AA |  |  | Throback et al (2004) |
| nosZ | nosZ2F | CGC RAC GGC AAS AAG GTS MSS GT | 65-60 (TouchDown) | 257 | Henry et al 2006 |
|  | nosZ2R | CAK RTG CAK SGC RTG GCA GAA |  |  |  |
| qnorB | qnorB2f | GGN CAY CAR GGN TAY GA | 55 | 263 | Kim 2020 |
|  | qnorB5r | ACC CAN AGR TGN ACN ACC CAC CA |  |  |  |
| Fungal nirk | nirKfF | TACGGGCTCATGTAYGTNSARCC | 54 | 480 | Wei et al 2015 |
|  | nirKfR | AGGAATCCCACASCNCCYTTNTC |  |  |  |

**References**

Harter, J., Krause, H. M., Schuettler, S., Ruser, R., Fromme, M., Scholten, T., Kappler, A., & Behrens, S. (2014). Linking N₂O emissions from biochar-amended soil to the structure and function of the N-cycling microbial community. *The ISME Journal, 8*(3), 660–674. <https://doi.org/10.1038/ismej.2013.160>

Henry, S., Bru, D., Stres, B., Hallet, S., & Philippot, L. (2006). Quantitative detection of the *nosZ* gene, encoding nitrous oxide reductase, and comparison of the abundances of 16S rRNA, *narG*, *nirK*, and *nosZ* genes in soils. *Applied and Environmental Microbiology, 72*(8), 5181–5189. <https://doi.org/10.1128/AEM.00231-06>

Kim, H. (2020). Comparison of PCR primers for analyzing denitrifying microorganisms in the hyporheic zone. *Applied Sciences, 10*(12), 4172. <https://doi.org/10.3390/app10124172>

Leininger, S., Urich, T., Schloter, M., Schwark, L., Qi, J., Nicol, G. W., ... & Schleper, C. (2006). Archaea predominate among ammonia-oxidizing prokaryotes in soils. *Nature, 442*(7104), 806–809. <https://doi.org/10.1038/nature04983>

Michotey, V., Mejean, V., & Bonin, P. (2000). Comparison of methods for quantification of cytochrome cd(1)-denitrifying bacteria in environmental marine samples. *Applied and Environmental Microbiology, 66*(4), 1564–1571. <https://doi.org/10.1128/AEM.66.4.1564-1571.2000>

Rosch, C., Mergel, A., & Bothe, H. (2002). Biodiversity of denitrifying and dinitrogen-fixing bacteria in an acid forest soil. *Applied and Environmental Microbiology, 68*(8), 3818–3829.

Rotthauwe, J. H., Witzel, K. P., & Liesack, W. (1997). The ammonia monooxygenase structural gene amoA as a functional marker: Molecular fine-scale analysis of natural ammonia-oxidizing populations. *Applied and Environmental Microbiology, 63*(12), 4704–4712.

Treusch, A. H., Leininger, S., Kletzin, A., Schuster, S. C., Klenk, H. P., & Schleper, C. (2005). Novel genes for nitrite reductase and Amo-related proteins indicate a role of uncultivated mesophilic crenarchaeota in nitrogen cycling. *Environmental Microbiology, 7*(12), 1985–1995. <https://doi.org/10.1111/j.1462-2920.2005.00906.x>

Throback, I. N., Enwall, K., Jarvis, Å., & Hallin, S. (2004). Reassessing PCR primers targeting nirS, nirK and nosZ genes for community surveys of denitrifying bacteria with DGGE. *FEMS Microbiology Ecology, 49*(3), 401–417. <https://doi.org/10.1016/j.femsec.2004.04.011>

Vanparys, B., Spieck, E., Heylen, K., Wittebolle, L., Geets, J., Boon, N., & De Vos, P. (2007). The phylogeny of the genus Nitrobacter based on comparative rep-PCR, 16S rRNA and nitrite oxidoreductase gene sequence analysis. *Systematic and Applied Microbiology, 30*(4), 297–308.

Wei, W., Kazuo, I., Yutaka, S., Tomoyasu, N., Nobuhito, O., Yuta, I., Shigeto, O., & Keishi, S. (2015). Development of PCR primers targeting fungal nirK to study fungal denitrification in the environment. *Soil Biology and Biochemistry, 81*, 282–286. <https://doi.org/10.1016/j.soilbio.2014.12.021>
